# Editorial: Modulating plasmacytoid dendritic cells: balancing cancer immunity and tumor progression

**DOI:** 10.3389/fimmu.2026.1954345

**Published:** 2026-08-18

**Authors:** Matilde Monti, Caroline Aspord, Claudio Tripodo, William Vermi

**Affiliations:** 1 Department of Molecular and Translational Medicine, University of Brescia, Brescia, Italy; 2 Univ. Grenoble Alpes, Inserm U 1209, Centre National de la Recherche Scientifique, Unité Mixte de Recherche (CNRS UMR) 5309, Institute for Advanced Biosciences, Grenoble, France; 3 Etablissement Français du Sang Auvergne-Rhône-Alpes, Research and Development (R&D) Laboratory, Grenoble, France; 4 The Associazione Italiana per la Ricerca sul Cancro - Italian Association for Cancer Research (AIRC) Institute of Molecular Oncology, Ente del Terzo Settore - Third Section Organization (IFOM-ETS), Milan, Italy; 5 Department of Oncology and Hematology-Oncology, University of Milan, Milan, Italy; 6 Unit of Pathology, “Azienda Socio‑Sanitaria Territoriale - Territorial Social and Health Authority (ASST) Spedali Civili di Brescia”, Brescia, Italy; 7 Department of Pathology and Immunology, Washington University School of Medicine, Saint Louis, MO, United States

**Keywords:** hematopoietic stem & progenitor cells (HSPCs), plasmacytoid dendritic cell (PDC), TLR7/9 pathway, tumor immunology, tumor microenvironment - TME, type I interferon (IFN)

Plasmacytoid dendritic cells (pDCs) are a unique subset of innate immune cells originating from the bone marrow (BM) (1). They are primarily known for their ability to produce type I interferons (I-IFNs) upon detecting nucleic acids (NAs) through endosomal Toll-like receptors (TLR)-7 and 9 (2). Recent studies have expanded our understanding of pDCs, revealing their capacity to sense cytosolic DNA via the cGAS-STING pathway, independent of TLR7/9, which also triggers potent I-IFN production (3–5). Single-cell RNA sequencing has recently revealed that pDCs adopt distinct tissue-specific and developmentally regulated transcriptional states (6), raising the possibility that analogous pDC functional states may emerge or be reshaped within the tumor microenvironment (TME). The role of pDCs in the context of cancer remains ambiguous (7). While they can contribute to cancer immunosurveillance through robust I-IFN responses, their presence in the TME is frequently associated with a tolerogenic phenotype, characterized by low I-IFN production and the promotion of regulatory T cells (8, 9). This suggests that the TME may induce a tumor-promoting functional state in pDCs (7, 10, 11). Despite these insights, the mechanisms underlying pDC activation and their potential therapeutic reprogramming in cancer remain underexplored, highlighting a significant gap in current research.

The articles collect in this Research Topic aim to elucidate the complex role of human pDCs in cancer immunity, focusing on their activation, function, and potential therapeutic modulation. Key objectives include understanding the heterogeneity of pDCs and identifying immune escape mechanisms induced by the TME in human cancers. The strategy is to move beyond the descriptive analysis of pDC dysfunction toward the active modulation and reprogramming of pDC differentiation and plasticity by deciphering their molecular determinants. The ultimate goal of the research is the transition from observing pDC failure to implementing therapeutic strategies that can restore pDC functions, disrupt the immunosuppressive TME and enhance anti-tumor immunity.

The review by Vasilakou et al. examined current knowledge and explore the mechanisms that restrain IFN-production by tumor-infiltrating pDCs, despite the accumulation of NAs deriving from extensive immunogenic cell death within the TME and the ability of pDCs to sense NA through TLR7/8 and 9. A key question is how the TME reprograms pDCs towards an immunosuppressive functional state and whether it is reversible. These mechanisms include alterations in TLR signaling, chronic stimulation and progressive pDC exhaustion, and limited accessibility of tumor-derived NAs. Particularly, the nature and immunostimulatory potential of tumor-derived NAs, and their intracellular delivery were discussed to identify strategies to restore the pDC–mediated antitumor immunity. In fact, impaired or actively restricted NA internalization limits pDC activation in cancer, but could be overcome by synthetic TLR ligands. Preclinical studies have shown that TLR7/8 and TLR9 agonists used as adjuvants of anti-cancer therapies can enhance anti-tumor immune responses and improve outcomes. *In situ* immunization with Vidutolimod through intratumoral injection can induce a systemic anti-tumor immune response and has shown promise in early phase cancer clinical trials (12). Vidutolimod is a nanosized virus-like particle (VLP) composed of the Qb bacteriophage capsid assembled around a TLR9 agonist. Activation of intratumoral pDCs plays a key role in initiating the anti-tumor response to Vidutolimod and is dependent on coating of the VLP by antibodies against the Qb capsid (anti-Qb). Lemke-Miltner et al. evaluated the mechanisms responsible for the binding, internalization and response of pDCs to anti-Qb-coated Vidutolimod. In most immune cells anti-Qb-coated Vidutolimod interaction is mediated by canonical Fcγ receptors, whereas in pDCs the uptake of anti-Qb-coated Vidutolimod was found to be mediated largely by BDCA2, a type II C-type lectin selectively expressed by pDCs, that has been recognized as an I-IFN inhibitory receptor. Of note, the concentrations of anti-Qb antibody coating Vidutolimod determined whether interaction of Vidutolimod with BDCA2 results in pDC activation or inhibition. Specifically, higher concentrations of anti-Qb induced significant internalization of BDCA2 and reduced activation of pDCs, viceversa pDC activation occurred when lower concentrations of anti-Qb were used. These mechanistic data could influence the design of future agents for *in situ* immunization based on activation of intratumoral pDCs.

The clinical implementation of pDC therapy has long been limited by the scarcity of pDCs in blood, their reduced functionality in cancer patients and their low viability during ex vivo manipulation. Overcoming these hurdles requires robust manufacturing protocols that produce *bona fide* pDCs (13–15). Two articles in this Research Topic has developed *in vitro* systems to produce surrogates for primary pDCs from CD34+ hematopoietic progenitors. Fiore et al. designed an *in vitro* differentiation protocol enabling the generation of large numbers of functional pDCs from human cord blood (CB) hematopoietic stem cells (HSCs) by using StemRegenin 1 (SR-1) and GM-CSF supplementation. CB-pDCs resemble primary pDCs phenotypically, transcriptionally and functionally, thus emerging as surrogates to investigate pDC pathophysiology and potential use as vectors for therapy. Of relevance, CB-pDCs were able to promote inflammation and anti-tumor immune responses in the TME. Specifically, in co-cultures with patient tumor explants, TLR7-treated CB-pDCs elicits cytokines associated with NK and T cell recruitment and potential cytolytic function, suggesting properly activated CB-pDCs as potent immunotherapeutic agents. A prerequisite for effective pDC-based immunotherapy is the resolution of the cellular heterogeneity inherent in both primary and *in vitro*-differentiated populations. Sanchez Hernandez et al. phenotypically and functionally characterized the *in vitro* hematopoietic stem and progenitor cells (HSPC)-pDCs heterogeneity. They identify three cell subsets, based on the expression of CD123 and BDCA2 surface markers, suggesting potential differences in their biological functions. Importantly, they identified the CD123^high^ population which shows the highest transcriptomic homology to blood-circulating pDCs and is the major producer of IFNα in response to TLR9 stimulation. By genetically manipulating the key master transcription factors (TFs) expression during HSPC-to-pDC differentiation, the authors identify a core transcriptional network associated with the pDC developmental trajectory that are essential to produce TLR9-responsive pDCs and to increase their frequency. These findings pave the way for reprogramming pDCs differentiation towards the IFNα-producing subset and their use as an immunomodulatory cell therapy.

The integration of pDC-based cell therapies with existing checkpoint blockades represents the next challenge in immuno-oncology. Collectively, the studies in this Research Topic illustrate complementary strategies that have the potential to reverse pDC-mediated tolerance and to strengthen immunosurveillance, and that are essential to translate preclinical insights into clinical benefit.
